# Prenatal ultrasound diagnosis of pentalogy of Cantrell: A case report

**DOI:** 10.1097/MD.0000000000043674

**Published:** 2025-08-08

**Authors:** Wentong Peng, Lijiao Li, Rongbi Yang, Hong Xie, Yi Lan

**Affiliations:** a Department of Ultrasound Medicine, Ziyang Hospital of Traditional Chinese Medicine, Ziyang, Sichuan Province, China.

**Keywords:** case report, congenital malformations, pentalogy of Cantrell, prenatal diagnosis, ultrasound

## Abstract

**Rationale:**

Pentalogy of Cantrell is a rare congenital condition characterized by a combination of 5 severe midline defects, including cardiac malformations, pericardial defects, sternal malformations, diaphragmatic defects, and abdominal wall defects.

**Patient concerns:**

A 31-year-old primigravida at 12 weeks of gestation visited our hospital in December 2022 for cough and expectoration.

**Diagnoses:**

Two-dimensional ultrasound revealed thoracoabdominal wall defects, an extrathoracic heart, liver displacement, and intestinal tubes protruding from the umbilicus, indicating key features of pentalogy of Cantrell.

**Interventions:**

After a multidisciplinary consultation on the malformation’s severity and treatment options, the family chose to terminate the pregnancy.

**Outcomes:**

A postmortem confirmed the ultrasound findings.

**Lessons:**

This case highlights the critical role of routine prenatal ultrasound examinations in detecting rare congenital malformations.

## 1. Introduction

Pentalogy of Cantrell (PC) is a rare and complex congenital condition, comprising 5 distinct midline birth anomalies, including defects of the heart, pericardium, diaphragm, sternum, and abdominal wall.^[1,2]^ To date, there have been approximately 250 reported cases of PC, with the majority of these occurring in the United States and Europe, representing 72% of documented cases.^[3]^ The incidence of PC is estimated to be between 1 in 65,000 and 1 in 200,000 live births, with a noted male predominance of 1.35:1.^[3,4]^

Prenatal diagnosis of PC can be achieved through 2D ultrasound as early as the first trimester, especially when a large omphalocele or ectopia cordis (EC) is present. However, it is more commonly diagnosed during the second trimester.^[3,4]^ When an external developmental defect is suspected, 3D ultrasound can provide a more detailed anatomical survey. Prenatal diagnosis allows families to make informed decisions regarding the continuation or termination of a pregnancy. This case report aims to highlight the role of prenatal ultrasound in the diagnosis of PC.

## 2. Case report

A 31-year-old primigravida female presented to the Obstetrics Department of Ziyang Traditional Chinese Medicine Hospital on December 19, 2022, with a 3-day history of cough, expectoration, rhinorrhea, and nasal congestion, in the absence of sore throat or fever. Her last menstrual period was documented as October 1, 2022. Neither of the couple has a congenital malformation family history, and they are in a non-consanguineous marriage. The patient had been administered dydrogesterone for a minor episode of vaginal bleeding on the 30th-day after the cessation of menstruation, effectively stopping the bleeding. No other abnormal drug use was reported. The measurement of nuchal translucency (1.97 mm) in the first trimester of 12 weeks’ gestation suggested a low risk of chromosomal anomaly.

**Figure 1.**
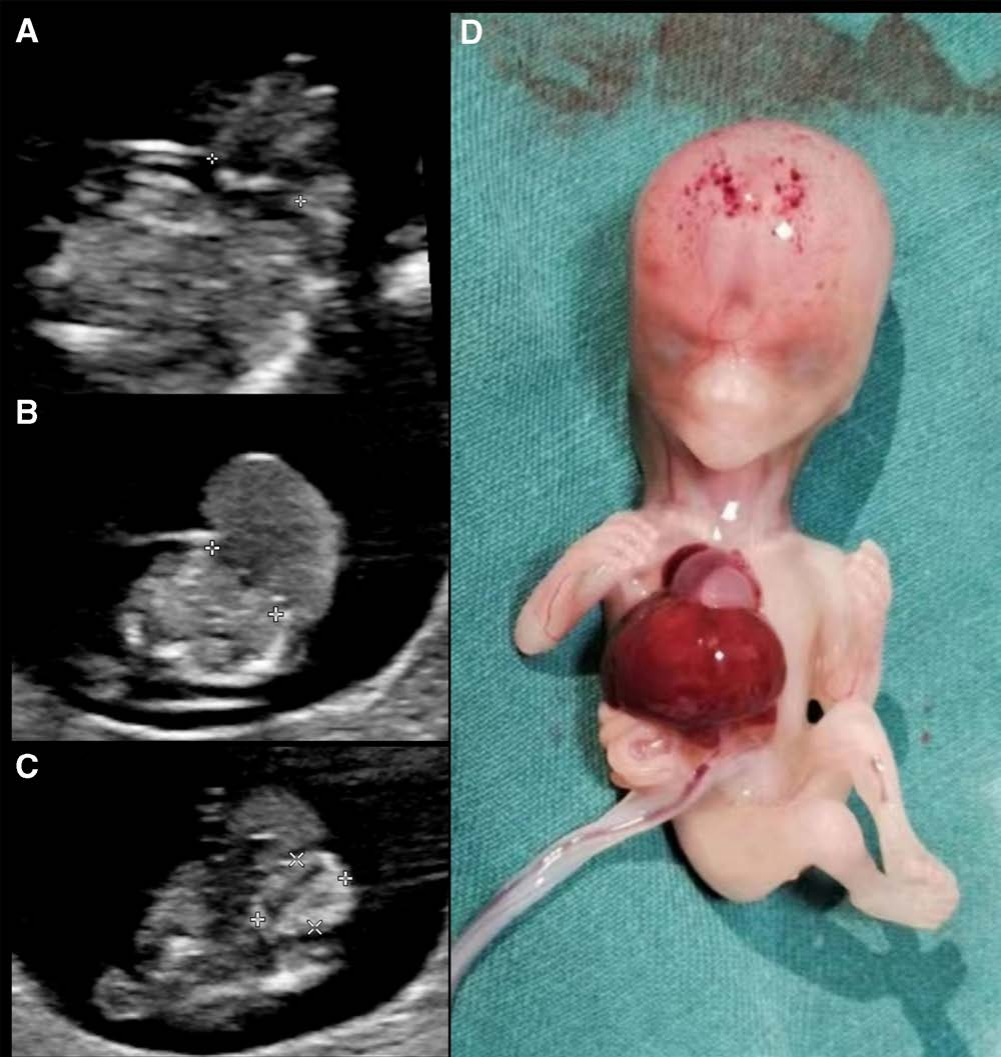
Imaging of pentalogy of Cantrell. (A) Fetal chest wall defect with exposed heart; (B) upper abdominal wall defect above the umbilicus with exposed liver; (C) fetal umbilical herniation; (D) the postnatal photograph following induced labor.

In our department, the 2D ultrasound revealed (Fig. 1A–C) an interruption in the continuity of echogenicity within the fetal thoracoabdominal wall, a defect in the lower part of the thoracic wall measuring approximately 5 mm in width, with the heart positioned externally to the thoracic wall. The heart’s pulsation was observed, and a 4-chambered heart was displayed. Additionally, there was a defect in the upper abdominal wall about 10 mm wide, with the liver displaced outside the abdominal wall, measuring approximately 15 × 8 mm. A portion of the intestinal tube was observed protruding from the umbilicus, measuring about 8 × 6 mm. These findings suggested a congenital malformation consistent with PC. Otherwise, ultrasound revealed herniation of abdominal contents through the umbilical ring, but with an intact umbilical cord and no connection or adherence with the amniotic cavity.

Multidisciplinary consultations were provided to the pregnant woman and her family, explaining the fetal malformation’s location, severity, potential post-birth impairments, and treatment options. After careful consideration, the family decided to terminate the pregnancy. The patient underwent labor induction at 12 weeks of gestation. The postmortem examination of the fetus revealed a large mass anterior to the thoracic and abdominal wall, with the heart, liver, and a portion of the intestinal tubes exposed without any membranous covering, aligning with the prenatal ultrasound findings (Fig. 1D), but no formal autopsy was performed due to the refusal of the family.

## 3. Discussion

This study presents a case of a prenatal diagnosis of PC, highlighting the significance of routine prenatal ultrasound examinations in detecting rare congenital diseases.

The fetal malformations observed in this case during routine ultrasound examination are consistent with PC. The pathogenesis of PC remains unclear, with prevailing hypotheses suggesting a potential etiology stemming from defective or mutated mesodermal migration during the early embryonic period (14–18 days). Additional contributing factors may include vascular dysplasia leading to blood stealing, mechanical factors leading to malformations secondary to amniotic rupture, tugging and adhesion, genetic mutations or viral infections in the third trimester, and maternal medication use.^[2,3,5–9]^ During early embryonic development, the fetal thoracoabdominal wall is formed by the fusion of the head-tail lateral folds. Defects in fold development can lead to thoracoabdominal wall fissures or protrusions. The extrusion of the heart from the thoracic cavity and thoracoabdominal wall defects are typical manifestations of PC. PC is frequently combined with endocardial malformations,^[4]^ with tetralogy of Fallot and ventricular septal defects being the most prevalent.^[1,3]^ Besides cardiac malformations, fetuses with PC often have scoliosis, limb deformities, fetal hydrops, cranial malformations (anencephaly, hydrocephalus, exencephaly), cleft lip and palate, umbilical cord, and colloid anomalies.

PC needs to be differentiated from other similar congenital anomalies such as body stalk anomaly, amniotic band syndrome, isolated EC, isolated abdominal wall defects, and cloacal exstrophy. Body stalk anomaly and PC have similar formation mechanisms, but differ in the location of abdominal wall defects.^[10]^ Amniotic band syndrome results from amniotic bands causing various deformities depending on their location, such as thoracoabdominal wall defects, constriction rings, edema, and amputation.^[11]^ Isolated EC and isolated abdominal wall defects lack the additional features of PC.^[12]^ Cloacal exstrophy involves lower abdominal wall defects and exposed intestines.^[13]^

Ultrasound is the most economical, convenient, safe, and effective noninvasive method for early prenatal diagnosis of PC.^[7]^ Diagnosis can be confirmed as early as 10 weeks of gestation by identifying the typical ultrasound features of EC and omphalocele. Routine prenatal ultrasound screening between 11 and 13 + 6 weeks can facilitate early detection and diagnosis, enabling early termination of pregnancy to avoid late-term induced labor, reducing maternal suffering, and providing important guidance for clinical management.

## 4. Conclusions

In conclusion, PC is a rare malformation that necessitates prompt recognition and decision-making. Prenatal ultrasound is an effective tool for the early detection of PC.

## Author contributions

**Conceptualization:** Wentong Peng, Lijiao Li.

**Data curation:** Wentong Peng, Lijiao Li.

**Investigation:** Wentong Peng, Lijiao Li, Yi Lan.

**Methodology:** Wentong Peng, Lijiao Li.

**Writing – original draft:** Wentong Peng, Lijiao Li, Rongbi Yang, Hong Xie, Yi Lan.

**Writing – review & editing:** Wentong Peng, Lijiao Li, Rongbi Yang, Hong Xie, Yi Lan.

## References

[1] Cantrell JR Haller JA Ravitch MM. A syndrome of congenital defects involving the abdominal wall, sternum, diaphragm, pericardium, and heart. Surg Gynecol Obstet. 1958;107:602–14. 13592660

[2] Sana MK Rentea RM. Pentalogy of Cantrell. StatPearls. Treasure Island (FL) with ineligible companies. Disclosure: Rebecca Rentea declares no relevant financial relationships with ineligible companies. 2025.

[3] Berarducci J Serrano-Roman J Armenta-Moreno JI Cano-Zarate R Espinola-Zavaleta N. Pentalogy of Cantrell. CJC Pediatr Congenit Heart Dis. 2022;1:198–9. 37969932 10.1016/j.cjcpc.2022.06.003 PMC10642150

[4] Williams AP Marayati R Beierle EA. Pentalogy of cantrell. Semin Pediatr Surg. 2019;28:106–10. 31072457 10.1053/j.sempedsurg.2019.04.006 PMC6559797

[5] Yang L. Prenatal ultrasonic diagnosis of incomplete pentalogyof cantrell: case report. Chin J Med Imag Technol. 2011;27:2564–5.

[6] Kubba T Khalil A Abu-Rustum R. Prenatal diagnosis of pentalogy of Cantrell at 11-13 weeks: evidence for a hexalogy. J Obstet Gynaecol. 2013;33:85–6. 23259889 10.3109/01443615.2012.730079

[7] Yang TY Tsai PY Cheng YC Chang FM Chang CH. Prenatal diagnosis of pentalogy of cantrell using three-dimensional ultrasound. Taiwan J Obstet Gynecol. 2013;52:131–2. 23548235 10.1016/j.tjog.2013.01.017

[8] Li S. Prenatal ultrasonograpic diagnosis of fetal anomalies. Beijing: People’s Military Surgeon; 2004.

[9] Hou YJ Chen FL Ng YY. Trisomy 18 syndrome with incomplete cantrell syndrome. Pediatr Neonatol. 2008;49:84–7. 18947004 10.1016/S1875-9572(08)60018-2

[10] Sepulveda W Wong AE Simonetti L Gomez E Dezerega V Gutierrez J. Ectopia cordis in a first-trimester sonographic screening program for aneuploidy. J Ultrasound Med. 2013;32:865–71. 23620329 10.7863/ultra.32.5.865

[11] Singh AP Gorla SR. Amniotic Band Syndrome. StatPearls. Treasure Island (FL) ineligible companies. Disclosure: Sudheer Gorla declares no relevant financial relationships with ineligible companies. 2025.

[12] Revels JW Wang SS Nasrullah A. An algorithmic approach to complex fetal abdominal wall defects. AJR Am J Roentgenol. 2020;214:218–31. 31714849 10.2214/AJR.19.21627

[13] Prefumo F Izzi C. Fetal abdominal wall defects. Best Pract Res Clin Obstet Gynaecol. 2014;28:391–402. 24342556 10.1016/j.bpobgyn.2013.10.003

